# SEM-EDS-Based Elemental Identification on the Enamel Surface after the Completion of Orthodontic Treatment:* In Vitro* Studies

**DOI:** 10.1155/2016/7280535

**Published:** 2016-09-27

**Authors:** Monika Machoy, Julia Seeliger, Mariusz Lipski, Anna Wójcicka, Tomasz Gedrange, Krzysztof Woźniak

**Affiliations:** ^1^Division of Orthodontics, Pomeranian Medical University in Szczecin, Szczecin, Poland; ^2^Division of Orthodontics, Technical University Dresden, Dresden, Germany; ^3^Department of Preclinical Conservative Dentistry and Preclinical Endodontics, Pomeranian Medical University in Szczecin, Szczecin, Poland; ^4^Institute of Technology, Pedagogical University of Cracow, Cracow, Poland

## Abstract

Braces as foreign bodies in the mouth carry a risk of side effects and toxicity to the human body. This article presents the results indicating the possible toxic effects of tools used for cleaning the enamel after the completion of orthodontic treatment. The studies were carried out* in vitro*. The procedure of enamel etching, bonding orthodontic metal brackets, and enamel cleaning after their removal was performed under laboratory conditions. The enamel microstructure and elements present on its surface were evaluated using the scanning electron microscope (SEM). Silicon and aluminium were found in addition to the tooth building elements.

## 1. Introduction

Fixed orthodontic braces are increasingly being used to treat malocclusion in adolescents and adults. Clinical evaluation of the long-term presence of braces as foreign bodies in the mouth shows that they can be harmful to human health. Mucosal ulceration, acute irritation, and allergy to nickel and other alloying elements of orthodontic brackets and arches, mainly in the brazed components, caused by orthodontic braces can have damaging effects, both local and general [1–7].

The possible impact on human health of orthodontic resins and composite materials as well as their residues left on the tooth surface must be also taken into consideration [8]. All the materials used in dentistry have European authorization and overall safety certification. However, it does not exclude the possibility of cases of a negative impact of dental materials on health during their long-term presence in the mouth, depending on the sensitivity of individuals. Another stage, which can lead to exposure of the body to harmful materials, is the moment of cleaning the tooth surface from composite remnants. During the process of cutting or polishing, chemical elements are released from both cleaned and cleaning elements, which within the water spray allows for their immediate absorption through mucosa well supplied with blood and creates the risk of aspiration into the airways or ingestion of potentially harmful dust generated during cleaning. Teeth are typically cleaned with instruments comprising metallic alloys, which may contain heavy metals such as chromium, copper, lead, nickel, and zinc [9]. These metals bind with proteins in the body, replacing the naturally occurring macro- and microelements and causing dysfunction of cells. Therefore, they are toxic to them. Published studies have shown that damage to the oxidation of biological macromolecules results from binding of heavy metals with the protein of DNA and nucleotides [10]. Although it is known that metals, most of all aluminium, have a number of adverse health effects [11, 12], which persist for a longer period of time during and after exposure; the frequency of their use continues to grow. Their toxicity is a growing problem, affecting the evolution, nutrition, and environment [13–16]. Therefore, in the present study, it was decided to examine the enamel surface after the completion of orthodontic treatment for the presence of elements recognized as toxic to the human body.

## 2. Material and Methods

The studies were carried out* in vitro*. The material consisted of 15 premolars extracted for orthodontic reasons. The following conditions defined the exclusion criteria: the presence of developmental defects of enamel, that is, hypoplasia, turbidity or discoloration, caries, and fillings on the vestibular surface.

The selected teeth were stored for 30 days in demineralised water, with a thymol crystal (0.1%) at room temperature.

Prior to the bonding of orthodontic brackets, tooth surfaces were cleaned using a polisher (Top Dental, Poland) with fluoride-free toothpaste Pressage (Shofu Inc., Japan) designed to prepare the enamel before fixing orthodontic steel brackets (Dentaurum, Germany). Then, the teeth were washed with distilled water and dried with compressed air for 15 seconds. For fastening brackets, orthodontic composite material Transbond™ XT Light Cure Adhesive (3M Unitek, USA) was used, which required prior preparation of the enamel surface. The vestibular surface of the teeth was etched for 30 seconds with 37% phosphoric acid, Blue-Etch (CERKAMED, Poland), rinsed with distilled water for 15 seconds and dried with compressed air. The dental adhesive OptiBond Solo Plus (Kerr, USA) was rubbed with an applicator into the etched enamel surface for 15 seconds. Then, the surface was dried with a gentle stream of air for 3 seconds and cured with halogen curing lamp light of the intensity of 750 mW/cm^2^ for 20 seconds. The composite material Transbond XT Light Cure Adhesive was placed on the bracket surface. The bracket was pressed onto the enamel surface with commonly used orthodontic bracket tweezers. The orthodontic bracket was placed at the centre of the mesial-distal axis of the tooth, moving its centre 3.5 mm away from the edge of the occlusal surface. The distance was measured using an orthodontic positioner. Once the bracket was properly placed, the material was subjected to polymerization with halogen curing lamp light for 40 seconds.

The teeth with fixed orthodontic brackets were stored in demineralised water at room temperature for 24 hours. After this time, the brackets were removed mechanically with the ix827 pliers (IxionInstruments, USA) designed for removing all types of brackets.

The remains of the adhesive were removed from the surface using a micromotor, standardly mounted in a dental unit, at a speed of 40 000 revolutions/min with water cooling and pressure force of 1.0 N. Abrasive processing was applied using cup shaped polishers made of aluminium oxides and bonding silicone.

The enamel cleaning procedure was considered to be finished on the basis of the naked eye evaluation, without additional zooming, and touching with a dental probe in the unit lamp light. The assessment criteria were the smoothness of the tooth surface and the absence of residual composite material. After cleaning the surface, the tooth was washed with water spray using the air and water compressor attached to the dental unit.

Before treatment and after finishing the above cleaning procedure, the tooth surface microstructure was evaluated using the JEOL JSM6610LV scanning electron microscope with SEI and BEI detectors. Oxford's EDS (Energy-Dispersive X-ray Spectroscopy) was used to identify the elements, while data were analyzed in the Aztec Software. Microscopic studies were performed at the Institute of Technology of the Pedagogical University of Cracow. The analysis was performed in order to observe composite material remains on the enamel surface and evaluate other elements present on its surface and the surface of the tooth crown. The JEOL JSM6610LV scanning electron microscope is adapted to analyze highly developed surfaces. The microscope has high resolving power of at least 15 nm at a large depth of field, so it is possible to map the surface details of the test samples greatly enlarged. The test samples do not require special preparation of the surface, since the microscope is equipped with the so-called low vacuum allowing for observation of nonconductive specimens without the necessity of applying the conductive layer. The specimen was placed in the microscope chamber and then subjected to an electron beam generated by an electron gun and accelerated in an electric field of 1/30 kV. There is a deflection system in the path of the electron beam—deflection of the beam in the direction X-X and in the direction perpendicular thereto Y-Y. Then, the electron beam having typically a diameter of 10/200 nm moves to the specimen surface and is focused on it. The reaction of the beam with the observed specimen results in activation of electrons from its subsurface area, which are then trapped by the detector placed in the microscope chamber. The test specimens were analyzed using the secondary electron imaging (SEI) method, analysis of backscattered electron imaging (BEI), and EDS analysis. X-ray microanalyzer (EDS) enabled analyzing the chemical composition of the test sample in the selected micro area of its surface—in this case within the crown and the composite material.

Qualitative and quantitative analyses were made using a microprobe. The quantitative analysis involved plotting a graph of the distribution of elements along a specified line. The qualitative analysis shows the arrangement of the elements on the surface, wherein the amount of a given element is proportional to the brightness of the image in a given place.

The obtained results were subjected to statistical analysis, assessing the median of the set which was Q2 = 0.4. In order to assess the correlation between saccadic and qualitative variables, the Kruskal-Wallis test and *U* Mann Whitney test were used. When verifying the hypotheses, the level of significance was *p* = 0.05.

## 3. Results

**Figure 1:**
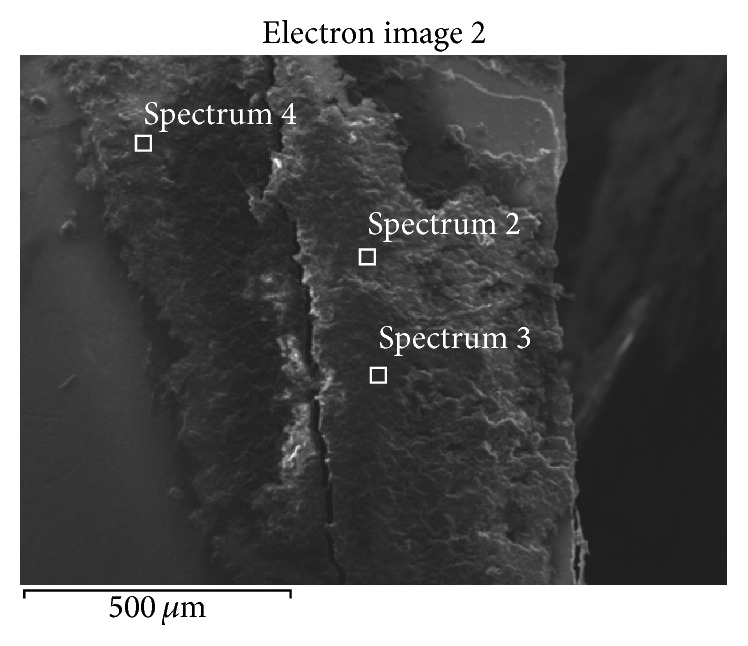
The residue of adhesive composite resins on the enamel surface left after tooth cleaning. Spectrums 2, 3, and 4 show the location of EDS analysis.

SEM-EDS analysis of the teeth surface after finishing the enamel cleaning procedure showed that although the operator considered the tooth surface to be completely clean and smooth, there were still composite resin residues on the enamel surface. Elemental analysis of the material residues and the completely cleaned tooth surface revealed that, in both cases, in addition to the naturally occurring elements building the tooth tissue which were transferred from the enamel surface to the composite material during cleaning (oxygen, carbon, hydrogen, nitrogen, calcium, phosphorus, sodium, and potassium), there occur also other elements which do not build the tissue: silicon and aluminium oxides.  Figure 1 shows an example of SEM image of the composite resin residues and the location of EDS analysis.

**Figure 2:**
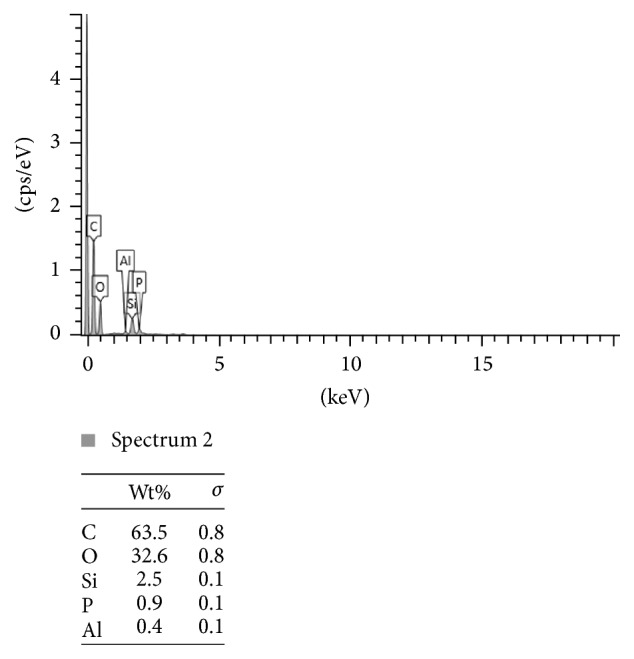
The SEM-EDS analysis of spectrum 2 showed in Figure 1. The percentage range of the elements in the composite mass, visualized in the adequate voltage range, counted in seconds per electron-volt. keV: accelerating voltage range used for EDS analysis, kilo-electron-volt. cps/eV: counts per second per electron-volt.

**Figure 3:**
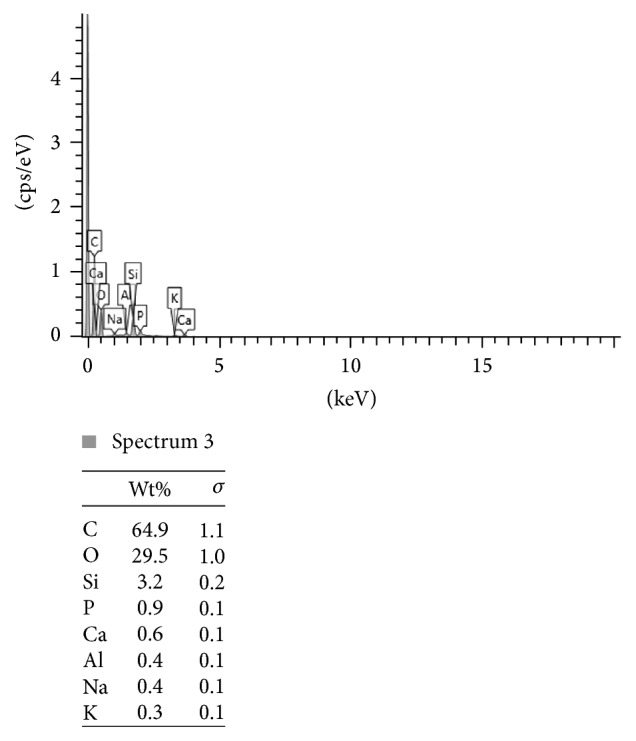
The SEM-EDS analysis of spectrum 3 showed in Figure 1. The percentage range of the elements in the composite mass, visualized in the adequate voltage range, counted in seconds per electron-volt. keV: accelerating voltage range used for EDS analysis, kilo-electron-volt. cps/eV: counts per second per electron-volt.

**Figure 4:**
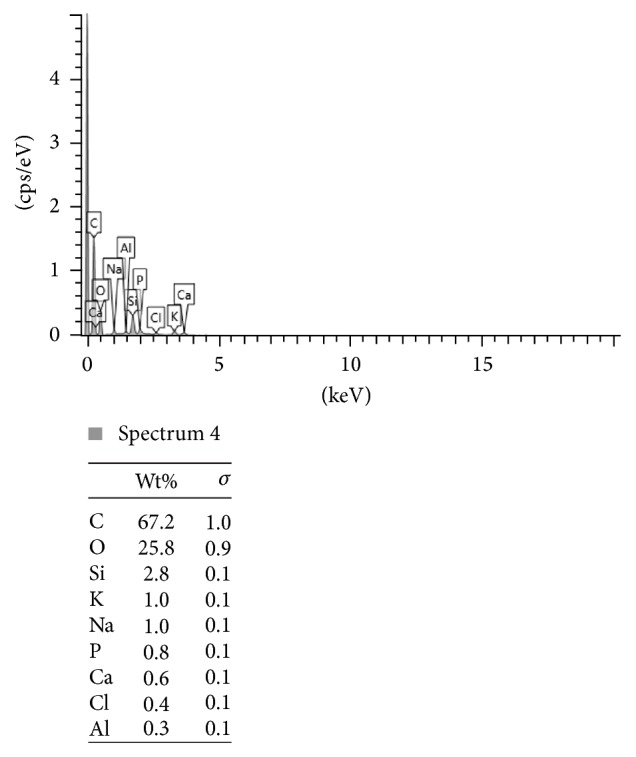
The SEM-EDS analysis of spectrum 4 showed in Figure 1. The percentage range of the elements in the composite mass, visualized in the adequate voltage range, counted in seconds per electron-volt. keV: accelerating voltage range used for EDS analysis, kilo-electron-volt. cps/eV: counts per second per electron-volt.

Figures 2, 3, and 4 present the example of the SEM-EDS analysis of spectrums 2, 3, and 4 showed in Figure 1. It shows the percentage range of the elements in the composite mass, visualized in the adequate voltage range, counted in seconds per electron-volt.

Table 1 shows the percentage of aluminium in the mass of orthodontic resin residues after completed enamel cleaning using silicone polishers with aluminium oxides.  Table 2 shows the percentage of aluminium on the enamel surface in the beginning of the treatment. There were no aluminium ions on the enamel in the control group.

The difference between the two groups was statistically significant (*p* < 0,05).

## 4. Discussion

Aluminium is ubiquitous on the Earth's surface. It is a metal which is light and corrosion-resistant [17]. Because of these properties, aluminium has become widely used in daily life. However, its soluble form produced by the industry is a potential health hazard for the living organisms [18]. Nowadays, people are exposed to it through food, which contains chemical additives, cooking in aluminium pots, packaging lined with aluminium, some preparations shielding the digestive tract and neutralizing gastric acid, and water, to which aluminium salts are added in the purification process. Aluminium interacts with most physical and cellular processes in humans. The exact mechanism of aluminium absorption by the gastrointestinal system is not yet fully understood. Based on the available literature, it is difficult to give the exact period of time for the toxicity of aluminium, because some of its symptoms are detected within a few seconds and others within a few minutes after exposure [19]. The toxicity of aluminium is probably the result of the interaction between apoplastic and symplastic objects [20]. In the human body, Mg^2+^ and Fe^3+^ are replaced by Al^3+^, which causes numerous disorders in intercellular communication, cell growth, and secretory functions. The changes that are caused by aluminium in neurons resemble degenerative changes observed in Alzheimer's disease. So far, reports have been published on the dissolution of metal ions from orthodontic braces and other dental materials in artificial saliva [21–24]. However, the enamel after the completion of orthodontic treatment has not been evaluated in this respect. Therefore, the comparison of the obtained results is not possible. In the present study, the most important and surprising observation is the presence of heavy metal ions on the enamel surface which was clinically assessed as cleaned. Qualitative assessment, which indicates the issue that needs to be examined, is more important than quantitative assessment.

The SEM-EDS analysis showed also the presence of silicone as a residue on the enamel after the orthodontic treatment. As silicone carcinogenicity end enzymes-inductive effects in mammals are lately brightly discussed and investigated [25, 26] it is very important to extend the researches in the dental industry. The silicones are regularly used in dentistry especially in endodontics as sealants, in prosthetics and orthodontics as impression materials, and in craniofacial surgery as internal implants. If the long-term studies give proof of silicone toxicity in human organisms it will be essential to change the whole methodology of treatment in many fields of medicine.

## 5. Conclusions

Metal ions, including aluminium, are present on the enamel surface after the completion of orthodontic treatment. The presence of aluminium was detected after cleaning the enamel using a polisher with aluminium oxides. Other methods for cleaning the enamel after orthodontic treatment should be assessed for the presence of residues of aluminium and other heavy metals. The problem of exposure of patients to adverse effects of heavy metals during and after orthodontic treatment should be explored and thoroughly investigated because of their potentially harmful effects on human health.

## Figures and Tables

**Table 1:** The percentage of aluminium in the mass of orthodontic resin residues after completed enamel cleaning using silicone polishers with aluminium oxides. Wt%Al: percentage of aluminium by mass. SD: standard deviation.

Tooth number	Wt%Al	SD
1	0,4	0,1
2	0,3	0,1
3	0,5	0,1
4	0,4	0,2
5	0,4	0,2
6	0,1	0,1
7	0,3	0,1
8	0,4	0,1
9	0,5	0,2
10	0,4	0,1
11	0,3	0,1
12	0,2	0,1
13	0,4	0,1
14	0,5	0,2
15	0,3	0,1

**Table 2:** The percentage of aluminium in the mass of orthodontic resin residues before the orthodontic treatment. Wt%Al: percentage of aluminium by mass.

Tooth number	Wt%Al
1	0
2	0
3	0
4	0
5	0
